# The challenge of sepsis in older adults in the Emergency Department and intensive care units

**DOI:** 10.62675/2965-2774.20260407

**Published:** 2026-05-20

**Authors:** Mariana Dias, Margarida Sá Machado, João Gonçalves Pereira, Mariana Alves, Susana Mendes Fernandes

**Affiliations:** 1 Unidade Local de Saúde Santa Maria Serviço de Medicina Interna Lisboa Portugal Serviço de Medicina Interna, Unidade Local de Saúde Santa Maria - Lisboa, Portugal.; 2 Unidade Local de Saúde Santa Maria Serviço de Medicina Intensiva Lisboa Portugal Serviço de Medicina Intensiva, Unidade Local de Saúde Santa Maria - Lisboa, Portugal.; 3 Universidade de Lisboa Faculdade de Medicina Clínica Universitária de Medicina Intensiva Lisboa Portugal Clínica Universitária de Medicina Intensiva, Faculdade de Medicina, Universidade de Lisboa - Lisboa, Portugal.; 4 Unidade Local de Saúde Santa Maria Unidade de Ortogeriatria Lisboa Portugal Unidade de Ortogeriatria, Unidade Local de Saúde Santa Maria - Lisboa, Portugal.

## INTRODUCTION

**Figure 1:**
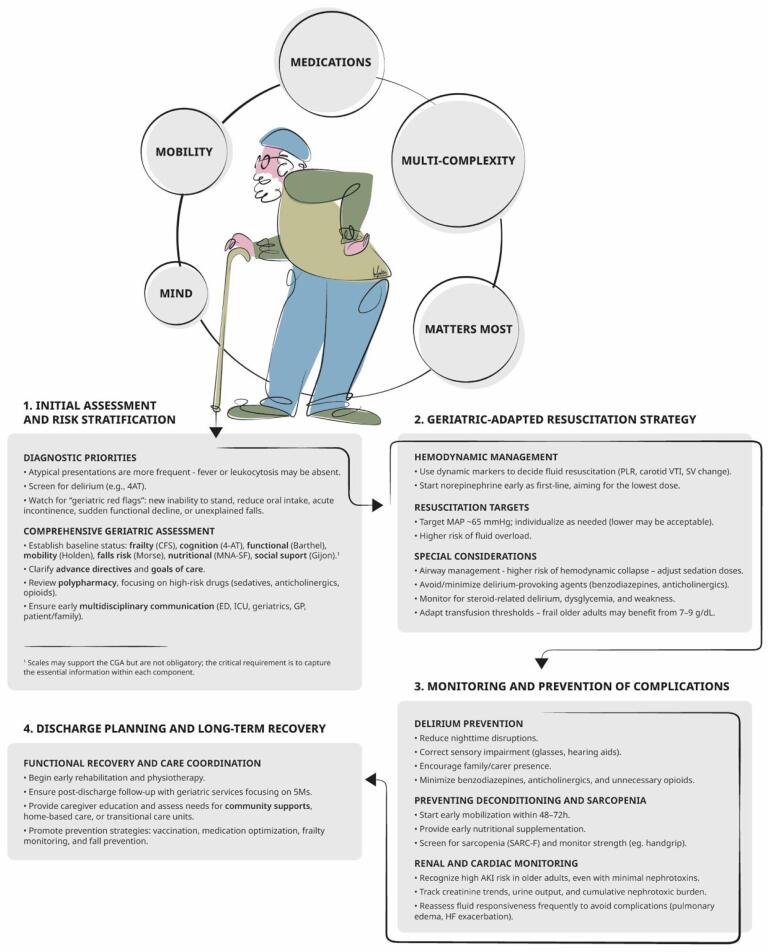
A geriatric pathway for sepsis: from vulnerability and diagnostic pitfalls to adapted bundles, frailty-triggered actions, and prevention.

Sepsis, a life-threatening organ dysfunction caused by a dysregulated host response to infection, affects individuals across all age groups.^(1)^ Still, older adults bear a disproportionate burden.^(2)^ Physiological changes associated with age, frailty, multimorbidity, and atypical presentations increase vulnerability. Despite this, Emergency Departments (ED) and intensive care units (ICU) remain poorly adapted to their needs.^(3)^ This viewpoint proposes a geriatric-based approach across the sepsis continuum to address these gaps (Figure 1).

## WHY ARE OLDER ADULTS MORE VULNERABLE TO SEPSIS?

Aging impairs innate and adaptive immunity ("immunosenescence"). It increases baseline inflammatory activation ("inflammageing"), reducing pathogen clearance, the ability to regulate systemic inflammation, and recovery after an acute insult, contributing to persistent organ dysfunction. Notwithstanding, understanding the immune system's aging process will lead to new prevention or treatment strategies, as recently reviewed.^(4)^

## WHAT ARE THE DETERMINANTS OF MISDIAGNOSIS OF SEPSIS IN OLDER INDIVIDUALS?

Classical signs such as fever and leucocytosis may be absent in older individuals, while nonspecific symptoms – delirium, weakness, anorexia, and acute functional decline – are easily misattributed to aging. Sepsis mimics, such as acute heart failure or pulmonary embolism, presenting with shock and elevated lactate, complicate sepsis recognition. Moreover, interpreting inflammatory biomarkers is difficult due to comorbidities, including chronic renal dysfunction.^(5)^ Ultimately, clinical acumen and structured geriatric assessment, understanding the role of altered mental state in the sepsis diagnosis, remain irreplaceable.

## MICROBIOLOGICAL DIAGNOSIS AND AGE-RELATED AETIOLOGIES

Causative pathogens shift with aging: *Gram*-negative bacilli such as *Escherichia coli* and *Klebsiella* spp., predominate, especially in urinary infections, whereas *Staphylococcus aureus* and *Streptococcus pneumoniae* remain important but less prevalent.^(6)^ Polypharmacy, indwelling devices, and prior healthcare exposure increase the likelihood of multidrug-resistant organisms or unusual pathogens.^(7)^ This highlights the need not to discard invasive microbiological sampling to guide antibiotic therapy.

## DO CURRENT BUNDLES FIT THIS POPULATION?

Although the Surviving Sepsis Campaign guidelines are foundational,^(1)^ most evidence has a limited representation of frail and older adults. A geriatric-based approach should adapt resuscitation, airway, and transfusion management to the older population's needs and focus on the 5Ms approach (*M*obility, *M*ind, *M*ulticomplexity, *M*edications, *M*atters Most)^(8)^ to promote an aggressive strategy for recovery and the prevention of complications (Figure 1).

## FLUID MANAGEMENT

In older adults, fluid tolerance may be reduced due to diastolic dysfunction, reduced renal function, and impaired autonomic regulation. Over 30mL/kg fluid loading may precipitate acute heart and kidney failure, cerebral edema, whereas conservative resuscitation risks hypoperfusion and multi-organ failure. As such, although initial fluids should not be delayed or withheld, monitoring based on ultrasound or even thermodilution methods should not be denied to this population to prevent both under- and over-resuscitation.^(9)^

## VASOPRESSOR THERAPY

Although norepinephrine remains first-line, this population is more susceptible to arrhythmias and myocardial ischemia at higher vasopressor doses. The OPTPRESS trial found no benefit from higher mean arterial pressure (MAP) targets (80 - 85mmHg), even suggesting potential harm in older hypertensive patients.^(10)^ In the 65-trial enrolling patients aged ≥ 65 years, reduced vasopressor exposure, aiming for MAP 60 - 65mmHg, was not associated with increasing mortality.^(11)^ These data reinforce that higher MAP goals should not be routinely pursued, and that a standard target around 65mmHg is appropriate, with adjustments in the presence of persistent signs of hypoperfusion.

## CORTICOSTEROID ADJUVANT THERAPY

Corticosteroid adjuvant therapy for refractory shock must be carefully considered. Overall, the risk of adverse events in older adults, including ICU-acquired weakness, dysglycemia, and neuro-psychiatric events, might be higher.^(12)^ Nevertheless, age analysis was not considered in recent systematic reviews.^(13)^

## TRANSFUSION THRESHOLDS

The optimal transfusion threshold in sepsis remains debated. Emerging evidence suggests that intermediate targets (Hemoglobin 7 - 9g/dL) may improve outcomes in older patients,^(14)^ eventually guided by the arterial-venous oxygen difference.^(15)^

## AIRWAY AND INTUBATION CONSIDERATIONS IN OLDER ADULTS

The indications for endotracheal intubation in sepsis include respiratory failure, hemodynamic collapse, altered mental status, and surgical source control. In the multicentre INTUBE study, age was an independent risk factor for peri-intubation cardiovascular instability or collapse.^(16)^ Anatomical and physiological changes associated with aging –reduced cervical spine mobility, diminished pharyngeal muscle tone, presbyphagia, and increased aspiration risk – compound the difficulty of airway management in older patients.^(17)^ These underscore the need to anticipate a difficult airway, optimize the hemodynamics before induction, and consider awake or modified induction strategies in older adults with sepsis.

## OPERATIONALIZING FRAILTY AT ADMISSION

Frailty assessment should be embedded in the sepsis admission evaluation, regardless of age. However, given the higher prevalence of frailty in older patients, the Clinical Frailty Scale (CFS), a nine-point tool that assesses global vulnerability, is particularly relevant for measuring baseline vulnerability. Importantly, frailty should not just be descriptive; it should guide concrete management steps. Such operationalization transforms frailty from a conceptual risk marker into a practical decision-making framework for acute sepsis care.^(18)^ Observational data confirm that frailty, rather than age alone, is the main predictor of ICU and hospital mortality even for the oldest old patients (more than 90 years old admitted to the ICU).

All patients with severe frailty (CFS ≥ 7) should have goals-of-care discussions, aligning treatment with patients’ values and a realistic prognosis. A multidisciplinary team approach, with ICU, geriatricians, and palliative care physicians, may provide further benefit.^(19)^

## BEYOND SURVIVAL: RECOVERY AND LONG-TERM SEQUELAE

Among survivors aged ≥ 65 years, sepsis often triggers a cascade of decline, new disabilities in activities of daily living, persistent cognitive impairment, institutionalization, and need for hospital readmission. These outcomes are mediated not only by the severity of the acute insult but also by baseline frailty and sarcopenia, as well as institutional standard of care.^(3)^ Strategies to prevent a fast decline after the event must be considered early on, such as rehabilitation to preserve mobility and *delirium* prevention bundles, minimizing the use of deliriogenic drugs, and maximizing the presence of family and caregivers. Although recommended for all patients, older adults may be perceived to have less benefit from being included in such an aggressive rehabilitation strategy.^(20)^

## CONCLUSION

The interplay of aging physiology, frailty, and multimorbidity shapes sepsis in older adults. Integrating a geriatric lens – grounded in frailty assessment, individualized hemodynamic stabilization, and recovery-focused care – offers a realistic, clinically meaningful path toward improved outcomes for older adults with sepsis, with a focus on functional recovery and quality of life.

## Data Availability

The contents underlying the research text are included in the manuscript.
